# Non-Esterified Fatty Acid-Induced Apoptosis in Bovine Granulosa Cells via ROS-Activated PI3K/AKT/FoxO1 Pathway

**DOI:** 10.3390/antiox12020434

**Published:** 2023-02-09

**Authors:** Zhiqi Lei, Ilyas Ali, Min Yang, Caixia Yang, Yifei Li, Lian Li

**Affiliations:** College of Animal Science and Technology, Nanjing Agricultural University, Nanjing 210095, China

**Keywords:** non-esterified fatty acid, ROS, apoptosis, steroidogenesis, PI3K/AKT signaling, FoxO1, bovine granulosa cells

## Abstract

Non-esterified fatty acid (NEFA), one of negative energy balance (NEB)’s most well-known products, has a significant impact on cows’ reproductive potential. Our study used an in vitro model to investigate the deleterious effects of NEFA on bovine granulosa cells (BGCs) and its underlying molecular mechanism. The results showed that high levels of NEFA led to the accumulation of reactive oxygen species (ROS), increased the expression of apoptosis-related factors such as Bcl2-Associated X/B-cell lymphoma-2 (Bax/Bcl-2) and Caspase-3, and down-regulated steroid synthesis-related genes such as sterol regulatory element binding protein 1 (SREBP-1), cytochrome P450c17 (CYP17), and cytochrome P450 aromatase (CYP19), to promote oxidative stress, cell apoptosis, and steroid hormone synthesis disorders in BGCs. In addition, NEFA significantly inhibited phosphatidylinositol 3-kinase (PI3K) and phosphorylated protein kinase B (p-AKT) activity and increased forkhead box O1 (FoxO1) expression. To further explore the role of the PI3K/AKT/FoxO1 signaling pathway in NEFA, we found that pretreatment with AKT-specific activator SC79 (5 mg/mL) for 2 h or transfection with FoxO1 knockdown siRNA in BGCs could alleviate the negative effects of NEFA treatment by decreasing Bax/Bcl-2 ratio and Caspase-3 expression, and upregulating SREBP-1, CYP17, and CYP19 expression. Meanwhile, SC79 significantly inhibited NEFA-induced dephosphorylation and massive nuclear translocation of FoxO1. Taken together, the NEFA induced oxidative stress, apoptosis, and steroid hormone synthesis disorders in BGCs by inhibiting the PI3K/AKT pathway that regulates FoxO1 phosphorylation and nuclear translocation. Our findings help to clarify the molecular mechanisms underlying the negative effects of high levels of NEFA on BGCs.

## 1. Introduction

The physiological state of dairy cows in the perinatal period is extremely special [1]. At this time, cows often experience a negative energy balance (NEB) due to insufficient dry matter intake and increased oxygen consumption by the tissues through cellular respiration [2]. The level of non-esterified fatty acid (NEFA) is an important index used to measure the NEB during the perinatal period [3], as NEB induces excessive fat mobilization which increases circulating NEFA concentrations. Under normal conditions, the basal concentration level of NEFA in bovine blood is 0.1 to 0.3 mM [4]. Due to the blood–follicle barrier, the concentration and composition of NEFA between blood and follicular fluid are slightly different, but the two are closely related, and the NEFA level in blood is reflected in follicular fluid [5]. When the circulating level of NEFA is higher than 0.75 mM, cows are susceptible to various diseases [6]. Furthermore, high levels of NEFA deposition in the organism are likely to result in peripartum dairy cattle suffering from oxidative stress [7]. When animals’ antioxidant clearance abilities are unable to counteract the production of reactive oxygen and nitrogen species (ROS, RNS) induced by high NEFA concentration, these free radicals exhibit negative effects on physiological processes, such as cell damage and dysfunction [8]. Numerous studies indicate that oxidative stress and apoptosis are closely linked in various animals, such as humans and mice [9]. ROS may activate cell apoptosis as a stress signal by activating or inhibiting various intracellular signaling pathways [10]. ROS accumulation, for instance, is what triggers NEFA-induced apoptosis in dairy cow hepatocytes [11]. However, the potential mechanisms and specific pathways by which high levels of NFEA affect oxidative stress and lead to apoptosis have not been elucidated to date.

Mammalian follicular atresia is a normal physiological process [12]. In point of fact, the vast majority of follicles are destined to undergo atresia during development, and only a small percentage of them are capable of maturing and eventually producing ovum [13]. Recent research has demonstrated a strong link between NEFA and ovarian development [14]. Oocyte development may be harmed by excess NEFA from NEB [5]. It is widely accepted that granulosa cell apoptosis is the primary factor that causes follicular atresia [15], and NEFA may act as a mediator between oxidative stress and apoptosis. Therefore, it is of great significance to focus on excess NEFA addition and related pathways of granulosa cell apoptosis for the growth and development of the follicle, as well as for the improvement of female mammalian fecundity.

It has been confirmed that the phosphatidylinositol 3-kinase (PI3K)/protein kinase B(AKT) signal pathway plays a crucial role in the regulation of granulosa cell growth and apoptosis during follicular development in a recent study [16]. This signaling pathway is basic transduction for the regulation of cell proliferation survival, migration, and metabolism in a variety of physiological and pathological processes [17]. Studies have shown that, in mice, hamsters, and humans, this signaling pathway can mitigate oxidative stress through the activation of downstream factors [18,19]. Forkhead box O1 (FoxO1) transcription factor is one of the important downstream target molecules of PI3K/AKT, which regulates multiple physiological processes [20,21]. A study in rats has noted that FoxO1 also has potential regulatory functions on follicular development [22]. Oxidative stress can trigger apoptosis of granulosa cells in mice by upregulating the expression of FoxO1 and, finally, leading to follicular atresia [23]. As a transcription-activating factor, the activity of FoxO1 is regulated by a variety of cytokines and signal pathways [24]. Among them, the specific amino acid site phosphorylation/dephosphorylation mediated by AKT is the chief mode of regulation [25]. In the absence of AKT-mediated phosphorylation, FoxO1 migrates to the nucleus due to dephosphorylation, resulting in enhanced transcriptional activity of apoptosis-promoting target genes [26]. In conclusion, significant progress has been made in recent years regarding the FoxO1 transcription factor. However, its mechanism of action through the PI3K/AKT pathway in follicles growth and development in cows with NEB is still poorly understood. At present, the specific functions and regulatory roles of the PI3K/AKT/FoxO1 pathway in NEFA-induced bovine granulosa cells (BGCs) require further investigation.

The purpose of this study was to investigate the effects and mechanisms of NEFA on BGCs. The role of the FoxO1 transcription factor in NEFA-induced oxidative stress and apoptosis in granulosa cells and its regulatory mechanism were investigated by adding PI3K/AKT activator SC79 and knocking down FoxO1. The results showed that NEFA negatively affected BGCs by inhibiting the PI3K/AKT pathway that drives FoxO1 into the nucleus to exert its transcriptional activity. Our study provided novel insights into how the PI3K/AKT signaling axis regulated FoxO1 under NEFA treatment, thus providing new ideas to further study the physiological state of cows in negative perinatal energy balance.

## 2. Materials and Methods

### 2.1. Primary Culture of Bovine Granulosa Cells

All procedures involving animals were approved in accordance with the guidelines of the Experimental Animal Welfare and Ethics Committee of the Nanjing Agricultural University (Approval number: SYXK(Su)2017-0027). Fresh bovine ovaries were collected from the local slaughterhouse in Nanjing, China, stored in a container with 37 °C normal saline, and sent to the study laboratory within 2 h. Upon arrival, ovarian samples were gently washed with normal saline, followed by washing for 30 s in prewarmed 75% ethanol. The ovaries were then immediately moved to the cell culture room for extraction after being washed three more times with normal saline to remove the blood. Subsequently, the follicular fluid was taken out of the follicle with a disposable needle, put in a 15 mL centrifuge tube, centrifuged for 5 min at 1000 rpm to collect the cells, washed two to three times with PBS, and centrifuged again to collect the cells. In this experiment, follicular fluid was extracted from healthy follicles between 3 and 6 mm in diameter, and at least 20 ovaries were obtained and mixed for the primary culture. Ultimately, the cells were then plated into T_25_ flasks (5 × 10^6^ cells/flask) in DMEM-F12 supplemented with 10% fetal bovine serum (FBS, FcMACS, Nanjing, China) and 200 U/mL of penicillin and streptomycin (Invitrogen, Carlsbad, CA, USA), and cultured at 37 °C in a humidified atmosphere containing 5% CO_2_. The medium was changed every 48 h.

### 2.2. Non-Esterified Fatty Acid Preparation

The NEFA stock (50 mM) solution contained oleic acid (21.75 mM, O1008, Sigma, St. Louis, MO, USA), linoleic acid (2.45 mM, L1376, Sigma, USA), palmitic acid (15.95 mM, P5585, Sigma, USA), stearic acid (7.2 mM, S4751, Sigma, USA), and palmitoleic acid (2.65 mM, P9417, Sigma, USA), which were dissolved with potassium hydroxide (0.1 M) at 60 °C, and the pH of the NEFA solution was adjusted to 7.4 using hydrochloric acid (1 M). To test the dose-dependent effect of NEFA in BGCs, the cells were seeded in 96-well plates and treated for different concentrations of NEFA (0, 0.3, 0.6, 0.9, 1.2, 1.5, 1.8, 2.1, and 2.4 mM) at 37 °C for 12 and 24 h. The optimum NEFA treatment concentration and processing time were selected and used for the subsequent experiment.

### 2.3. Cell Transfection and Treatment

For treatment with the unique specific AKT activator, SC79 (AbMole BioScience, USA) was dissolved in dimethyl sulfoxide (DMSO; Solarbio, Beijing, China) and stored at −80 °C. BGCs were treated for different concentrations of SC79 (0, 0.5, 1, 5, 10, 15, 20, 25 mg/mL) at 37 °C for 2 h before NEFA was added. The optimum SC79 concentration was selected and used for the subsequent experiment.

BGCs were plated on 6-well plates until they reached 60–80 percent of their final size. After that, the cells were infected for 6 h with siRNAs targeting bovine FoxO1 (FoxO1-siRNA/siFoxO1) and negative control siRNA (NC-siRNA) (GenePharma, Shanghai, China) using Lipofectamine 2000 reagent (Invitrogen, Carlsbad, CA, USA). The medium was changed until the cells were taken for analysis (The sense and antisense sequence of siFoxO1 are 5′- GGUGAAGACAGCUUUACAATT -3′, 5′- UUGUAAAGCUGUCUUCACCTT -3′; siNC promoter forward: 5′- UUCUCCGAACGUGUCACGUTT -3′; and si-NC promoter reverse: 5′- ACGUGACACGUUCGGAGAATT -3′).

### 2.4. Cell Viability Assay

The cell viability was detected using a cell counting kit-8 (CCK8; Jiancheng Bioengineering Institute, Nanjing, China). BGCs were seeded in 96-well plates for 12 h at 37 °C and 5% CO_2_ to a density of 70–80%. Following the specified treatments, the growth medium was removed from the cells, and PBS was used to wash them three times. After that, 10 μL CCK-8 solution was added to each well and incubated at 37 °C for 3 h. The absorbance was measured at 450 nm using a microplate reader. Cell viability was calculated as follows: cell viability (%) = (A_s_ − A_b_) / (A_c_ − A_b_) × 100% where A_s_, A_b,_ and A_c_ are the absorbance of sample, blank and negative control, respectively.

### 2.5. Immunofluorescence Staining

The cells were inoculated in 12-well plates containing cell slides. After the treatment, 4% paraformaldehyde was added to fix the cells at room temperature for 30 min and 0.5% Triton X-100 was added to permeabilize the cells for 20 min. After that, the cells were blocked with 5% BSA for 1 h, and then they were incubated with primary antibodies against FoxO1 (1:100; Cell Signaling Technology, MA, USA) for an overnight period at 4 °C. After the primary antibody was recovered and washed three times with PBS, FITC-conjugated secondary antibodies (1:100; Abcam, Cambridge, UK) were used to incubate the cells for 60 min in the dark. Finally, the nuclei were stained with diamidino-2-phenylindole (DAPI; KeyGEN, Nanjing, China) for 5 min. Images of the cells were taken using a confocal microscope (Zeiss LSM 700 META).

### 2.6. Assay for Malondialdehyde (MDA) and Superoxide Dismutase (SOD)

MDA and SOD levels were measured using colorimetric assay kits purchased from the Beyotime Institute of Biotechnology in Shanghai, China. The microplate reader measured the optical densities of MDA and SOD at 532 nm and 450 nm, respectively, in accordance with the manufacturer’s instructions.

### 2.7. Measurement of ROS

The intracellular ROS generation was detected using the 2,7-Dichlorofuorescin Diacetate (DCFH-DA; Jiancheng Bioengineering Institute, Nanjing, China) in accordance with the manufacturer’s directions. After being washed three times with PBS, the treated cells were stained for 60 min in the dark with 10 μM DCFH-DA at 37 °C before being examined under a fluorescence microscope (Olympus, Tokyo, Japan) at 500 and 525 nm.

### 2.8. Total RNA Extraction and Quantitative Real-Time Polymerase Chain Reaction (qRT-PCR)

To perform qRT-PCR, total RNA from BGCs was extracted using TRIzol regent (Invitrogen, Carlsbad, CA, USA). All procedures were performed on ice to prevent RNA degradation. The concentration and purity of RNA were detected by a NanoDrop 2000 spectrophotometer (Thermo Scientific, Waltham, MA, USA), and reverse transcription was performed with HiScript**^R^** III RT SuperMix (Vazyme, Nanjing, China). For qRT-PCR, the SYBR Green Master Mix (Vazyme, Nanjing, China) was used in accordance with standard protocols on ABI prism fast 7500 systems (Applied Biosystem, Foster, City, USA). An optimized comparative Ct (^2−ΔΔ^Ct) value method was used to normalize the gene expression data to that of β-actin after each sample was tested three times. The primer sequences and product lengths are presented in Table 1.

### 2.9. Preparation of Cytoplasm and Nuclear Extracts

Cytoplasm and nuclear extracts were prepared using the Nuclear and Cytoplasmic Protein Extraction Kit (KeyGEN, Nanjing, China). According to the manufacturer’s instructions, after being treated with the indicated drugs, cells were washed in ice cold PBS twice and then resuspended in 450 µL of ice cold cytoplasm extraction buffer A (every 1 mL buffer A with 17 μL 100 mM Phenylmethanesulfonyl fluoride (PMSF) and 1 μL protease inhibitor) and 50 µL cytoplasm extraction buffer B for 30 min. The cell lysates were centrifuged at 3000 rpm for 10 min at 4 °C, and the supernatant was aspirated as cytoplasmic protein and stored at −80 °C. Next, the nuclear pellets were resuspended in 100 µL ice cold cytoplasm extraction buffer C (every 1 mL buffer C with 17 μL 100 mM PMSF and 1 μL protease inhibitor). After 6 sets of vortexing for 15 s every 10 min on the ice, lysates were centrifuged at 14,000 rpm for 30 min at 4 °C. Nuclear extracts were aliquoted and stored at −80 °C until use.

### 2.10. Western Blot Analysis

Cells were lysed on ice with 200 μL Radio Immunoprecipitation Assay (RIPA; Beyotime, Shanghai, China) lysis buffer containing 1% proteinase inhibitor (PMSF; Beyotime, Shanghai, China). The cell lysate was centrifuged at 15,000 rpm for 15 min at 4 °C and the concentration of protein samples was determined using the BCA assay kit (Beyotime, Shanghai, China). Then, samples containing 50 μg protein were separated by 12% sodium dodecyl sulfate-polyacrylamide gels (SDS-PAGE; GenScript, Nanjing, China) and transferred to the polyvinylidene difluoride membrane (PVDF; Life Technologies Carlsbad, CA, USA). Membranes were blocked with 5% free-fat milk in Tris-buffered saline with tween (TBST; Solarbio, Beijing, China) for 2 h at room temperature and incubated with primary antibodies overnight at 4 °C (Table 2). After three washes with TBST, the membranes were incubated with HRP-conjugated secondary antibodies (Biosharp, Shanghai, China) for 2 h at room temperature. Finally, the signals were detected by the enhanced chemiluminescence substrate kit (ECL; Biosharp, Shanghai, China) and exposed to the chemiluminescence detection system (Amersham, Piscataway, Nanjing, China) following company guidelines. Immunoblots were scanned and densitometry was performed using ImageJ software (National Institutes of Health, Bethesda, MD, USA).

### 2.11. Flow Cytometer Detection of Apoptosis

The Annexin V-Alexa Flour 647/PI Apoptosis Assay Kit (FcMACS, Nanjing, China) was used to detect apoptosis in accordance with the manufacturer’s instructions. Following the above-mentioned procedures, the cells were seeded into six-well plates. The cells were washed with ice-cold PBS and then were gently removed using a brief trypsinization procedure (without EDTA; Gibco Life Technologies Carlsbad, CA, USA). After being centrifuged at 700 rpm for 10 min, the BGCs were stained for 15 min in the dark with 200 μL 1× annexin binding buffer containing 5 μL rh Annexin V-Alexa Flour 647 and 10 μL propidium iodide solution. The entire cell population can be analyzed for the proportion of apoptotic cells using flow cytometry with a FACS Calibur (BD Biosciences, Bedford, MA, USA), and analysis of the experimental data was carried out by using Flowjo software version 10.0.7 (Becton, Dickinson and Company, Franklin Lakes, JD, USA).

### 2.12. Statistical Analysis

Data were analyzed using the statistical software known as SPSS (version 20.0; SPSS Inc., Chicago, IL, USA) and expressed as mean ± SEM of three biological replicates. Statistical analysis was performed by one-way analysis of variance (ANOVA) followed by Tukey’s multiple comparison test between different groups. *p* value < 0.05, 0.01, 0.001 was considered statistically significant. The GraphPad Prism 6.01 software (GraphPad Software Inc., San Diego, CA, USA) was used to generate the graphs.

## 3. Results

### 3.1. Proliferative and Morphological Effects of NEFA Treatment on BGCs

**Figure 1:**
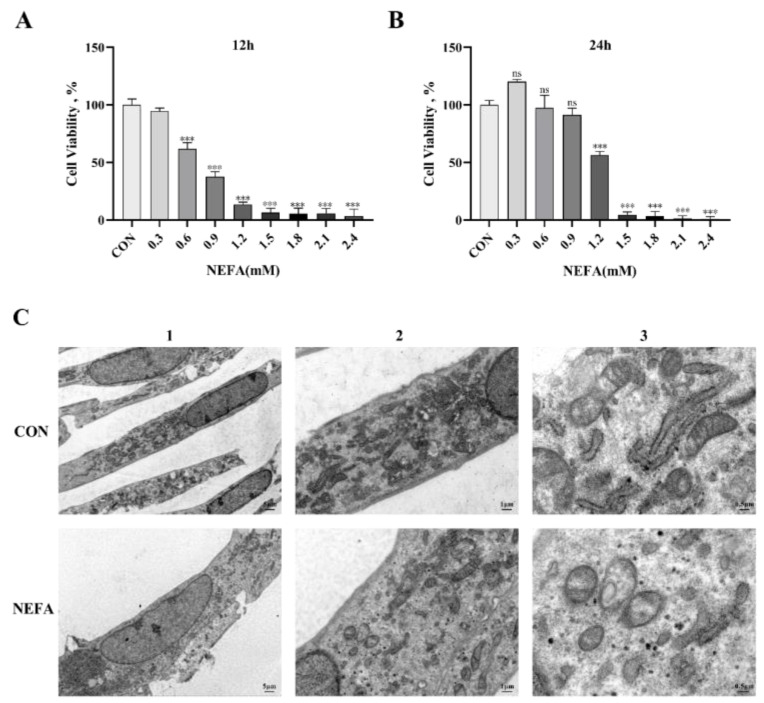
Non−esterified fatty acid (NEFA) treatment affected the growth and morphology of bovine granulosa cells (BGCs) in vitro. (**A**,**B**) Cell activity of BGCs was evaluated at different concentrations of NEFA supplementation for 12 and 24 h by performing a CCK−8 assay. (**C**) The morphology of BGCs treated with or without 1.2 mM NEFA for 24 h was observed by transmission electron microscopy: 1. Overall cell structure, scale bar: 5 µm; 2. Cell ultrastructure (local magnification of Figure 1(C1)), scale bar: 1 µm; 3. Observation of intracellular mitochondrial structure (local magnification of Figure 1(C2)), scale bar: 0.5 µm. This experiment was repeated 3 times, and data are expressed as mean ± standard errors of the mean (SEM), *** *p* < 0.001; ns, not significant.

In order to determine the optimal time and concentration of NEFA in BGCs and to prevent non-physiological cell death from excessive oxidation, cells were treated with various NEFA concentrations (0, 0.3, 0.6, 0.9, 1.2, 1.5, 1.8, 2.1, 2.4 mM) for 12 and 24 h. The CCK8 assay was then used to determine whether the cells were still viable. The findings demonstrated that as the amount of added NEFA increased, granulosa cell viability decreased in a concentration-dependent manner. At 1.2 mM NEFA concentrations, the cell viability decreased significantly (*p* < 0.001, Figure 1A,B) when the treatment duration was 24 h compared to the control group, reaching 56.312%. Accordingly, we picked 1.2 mM as the work focus and 24 h as the treatment time in the accompanying experiments. Furthermore, the transmission electron microscope was used to examine the morphology of the cells. The control group cell structure was complete, with clearly visible mitochondrial cristae and mitochondrial edges (Figure 1C). However, the mitochondria in the NEFA-treated group were vacuolated because they were smaller and had fewer cristae, and the cell membrane became incomplete.

### 3.2. NEFA Produced ROS and Induced Oxidative Stress in BGCs

**Figure 2:**
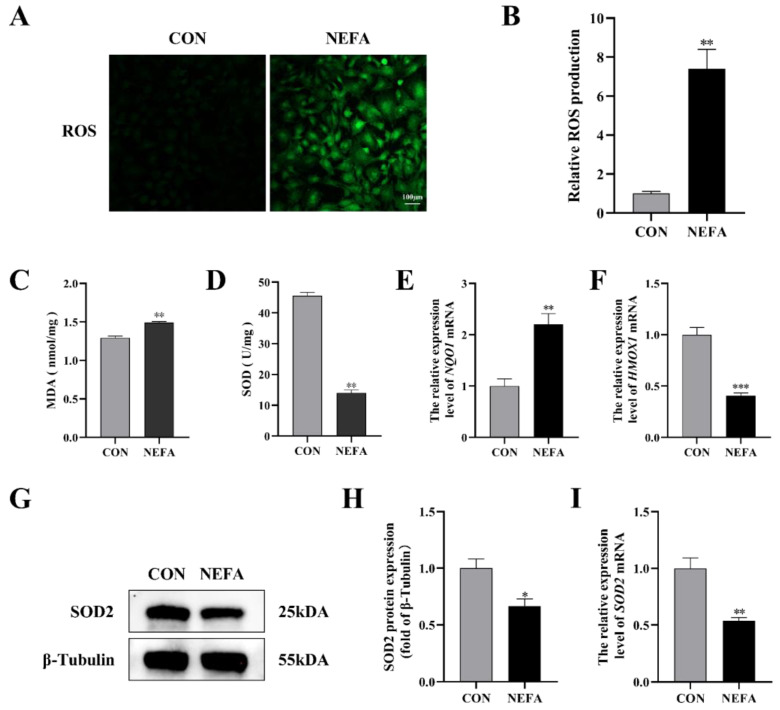
Effects of NEFA on ROS generation and oxidative stress in BGCs. BGCs were treated with or without 1.2 mM NEFA for 24 h. (**A**,**B**) Fluorescent microscopic images of ROS generation by using DCFH−DA staining and comparison of Relative fluorescence intensity between control and NEFA−treated. Green: DCFH−DA, scale bar: 100 µm. (**C**,**D**) The cellular MDA and SOD content was tested by Kits. (**E**,**F**) The mRNA levels of NQO1 and HMOX−1 were determined using qRT−PCR. (**G**–**I**) SOD2 protein and gene expression in BGCs. The quantification of the relative protein levels (performed using Image J) is shown under the band (protein/β−Tubulin). This experiment was repeated 3 times, and data are expressed as mean ± standard errors of the mean (SEM), * *p* < 0.05; ** *p* < 0.01; *** *p* < 0.001; ns, not significant.

There is sufficient evidence to suggest that an excessive accumulation of ROS is one of the primary factors that leads cells to oxidative damage and apoptosis. We found the total intracellular ROS level rose in the NEFA-treated group when compared to the control group (*p* < 0.01, Figure 2A,B). In order to further verify the effects of NEFA on cell oxidative stress, the activity and expression level of antioxidant enzymes were detected. When NEFA was added to the cells, MDA content increased significantly and SOD activity decreased significantly (*p* < 0.01, Figure 2C,D). In contrast to the control group, the NEFA group had higher levels of quinone oxidoreductase 1 (NQO1) mRNA and lower levels of heme oxygenase 1 (HMOX-1) mRNA and SOD2 expression, indicating a redox state imbalance (Figure 2E–I). Based on these findings, it appeared that NEFA may cause oxidative damage in BGCs.

### 3.3. NEFA-Induced Apoptosis in BGCs

**Figure 3:**
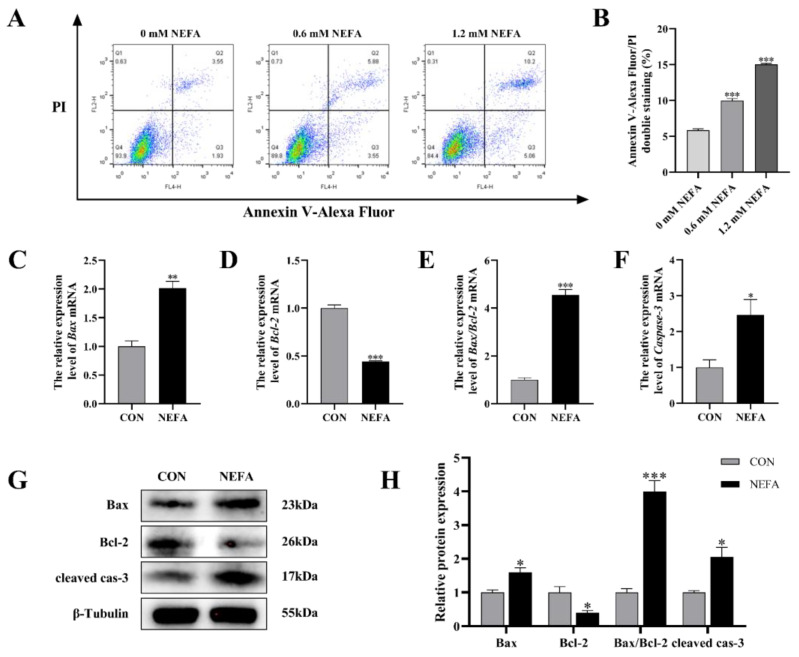
NEFA−induced apoptosis in BGCs. BGCs were treated with or without 1.2 mM NEFA for 24 h. (**A**,**B**) The apoptosis of BGCs which were treated with 0, 0.6, and 1.2 mM NEFA was determined by flow cytometry and analyzed using FlowJo. (**C**–**F**) The apoptosis−related mRNA levels of Bax, Bcl−2, and Caspase−3 were determined using qRT−PCR. (**G**,**H**) The apoptosis−related proteins Bax, Bcl−2, and Caspase−3 were analyzed by Western blot. The quantification of the relative protein levels (performed using Image J) is shown under the band (protein/β−Tubulin). This experiment was repeated 3 times, and data are expressed as mean ± standard errors of the mean (SEM), * *p* < 0.05; ** *p* < 0.01; *** *p* < 0.001; ns, not significant.

In order to explore the effects of NEFA on the apoptosis of BGCs, flow cytometry was used to detect cell apoptosis using Annexin V-Alexa Flour 647/PI double staining. As depicted in Figure 3A,B, the effect of NEFA on the apoptosis rate of cells was dose-dependent, and it was extremely significant in comparison to the control group (*p* < 0.001). To further confirm that NEFA influences apoptosis, we performed qRT-PCR and Western blot to measure the levels of apoptosis-related mRNA and protein expression of Bcl2-Associated X (Bax), B-cell lymphoma-2 (Bcl-2), and cleaved caspase-3. The NEFA-treated group had significantly more Bax and Caspase-3 mRNA expression and ratio of Bax/Bcl-2 than the control group (*p* < 0.05, Figure 3C–F), but, on the other hand, Bcl-2 levels exhibited the opposite trend. Western blot analysis also revealed similar results for the NEFA group in comparison to the control group (*p <* 0.05, Figure 3G,H). This finding demonstrated that BGCs could undergo apoptosis by NEFA.

### 3.4. Effects of NEFA Treatment on Steroid Hormone Synthesis Gene Expression in BGCs

**Figure 4:**
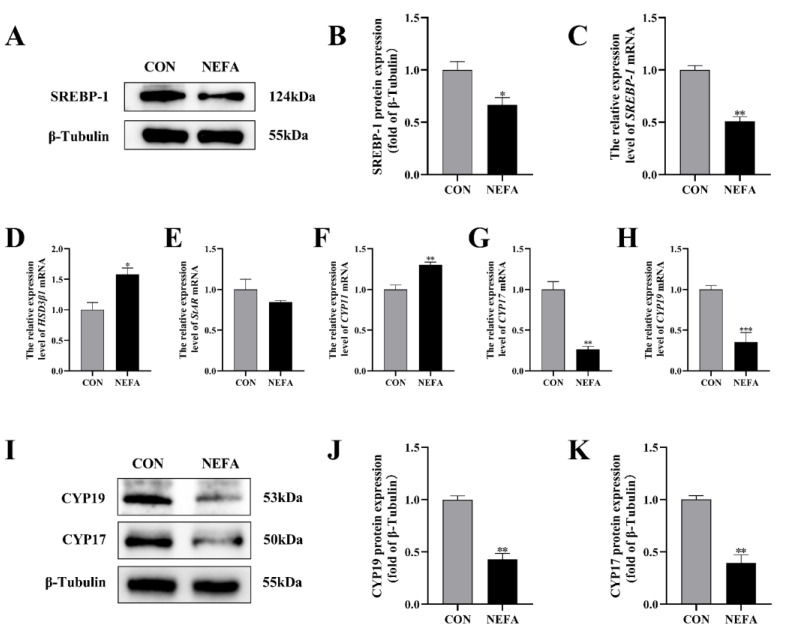
The effects of NEFA on the biosynthesis of transport and synthesis of steroid hormones in BGCs. BGCs were treated with or without 1.2 mM NEFA for 24 h. (**A**–**C**) SREBP−1 protein and gene expression in BGCs. (**D**–**F**) Effects of NEFA on gene expression levels on HSD3β1, StAR, and CYP11. (**G**–**K**) CYP17 and CYP19 protein and gene expression in BGCs. The quantification of the relative protein levels (performed using Image J) is shown under the band (protein/β−Tubulin). This experiment was repeated 3 times, and data are expressed as mean ± standard errors of the mean (SEM), * *p* < 0.05; ** *p* < 0.01; *** *p* < 0.001; ns, not significant.

In order to investigate the effects that NEFA has on the biosynthesis of steroid hormones in BGCs, we used qRT-PCR and protein blotting to determine the expression of the relevant genes. Sterol regulatory element binding protein 1 (SREBP-1) mRNA and protein levels were significantly lower in the NEFA-treated group than in the untreated control group (*p* < 0.05, Figure 4A–C). The addition of NEFA resulted in significant upregulation of cholesterol side chain cleavage enzyme (CYP11) and hydroxysteroid dehydrogenase 3β1 (HSD3β1), hindered the expression of steroidogenic acute regulatory protein (StAR) mRNA (Figure 4D–F), and caused a significant decrease in the level of cytochrome P450c17 (CYP17) and cytochrome P450 aromatase (CYP19) mRNA and protein following treatment (*p* < 0.01, Figure 4G–K). High levels of NEFA affected the expression of hormone-related genes in BGCs.

### 3.5. NEFA Regulated the Expression of PI3K/AKT Pathways in BGCs

**Figure 5:**
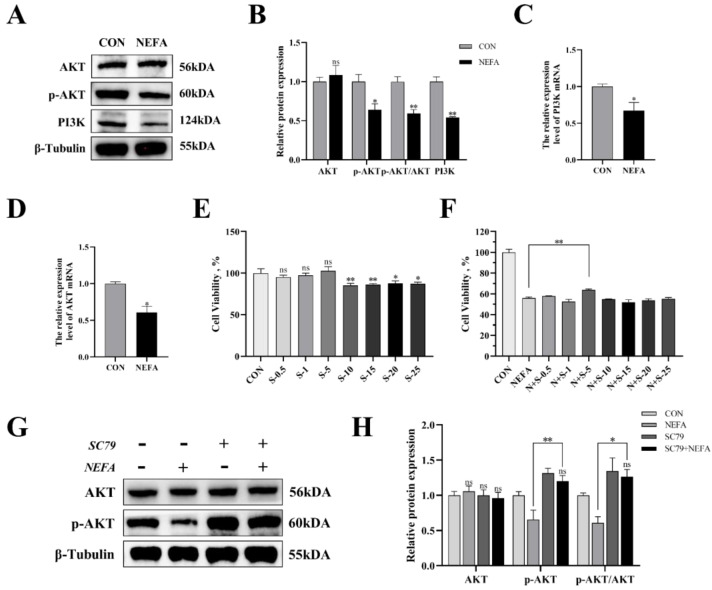
The effects of NEFA on the expression of PI3K/AKT pathways in BGCs and the concentration screening of pathway activator SC79. BGCs were treated with or without 1.2 mM NEFA for 24 h. (**A**–**D**) The protein and gene expression related to PI3K/AKT pathways in BGCs were determined by qRT−PCR assay and Western blot. (**E**) The cell activity of BGCs was measured using CCK−8 assay using different concentrations of SC79 for 2 h. (**F**) The cell activity of BGCs that were pretreated with different SC79 concentrations before NEFA treatment. (**G**,**H**) Detection of activation of AKT by 5 mg/mL SC79 with Western blot analysis. The quantification of the relative protein levels (performed using Image J) is shown under the band (protein/β−Tubulin). This experiment was repeated 3 times, and data are expressed as mean ± standard errors of the mean (SEM), * *p* < 0.05; ** *p* < 0.01; ns, not significant.

In this experiment, we examined the effects of NEFA on the PI3K/AKT signaling pathway of BGCs through immunoblotting and qRT-PCR. As demonstrated in Figure 5, treatment of the cells with NEFA significantly downregulated the expression of PI3K and p-AKT (*p* < 0.05); however, it did not change the AKT content (*p* > 0.05, Figure 5A–D). Hence, it could be speculated that NEFA altered the PI3K/AKT pathway in BGCs.

The experimental strategy made use of SC79, a one-of-a-kind, specific AKT activator, to further demonstrate the negative effects of NEFA on BGCs by inhibiting the aforementioned signaling pathways. First, we reported whether SC79 had any cytotoxic effects on BGCs. The viability of the cells was assessed with CCK8 at various concentrations of the SC79 pre-treatment prior to the addition with or without NEFA. The findings demonstrated that, within a certain concentration range, SC79 had no toxic effect on BGCs (*p* > 0.05, Figure 5E) and significantly alleviated the significant decrease in cell viability after NEFA treatment (*p* < 0.05, Figure 5F). This indicated that SC79 could at least partially restore the negative effects of BGCs caused by NEFA addition. Next, the effect of SC79 (5 mg/mL) treatment on the expression of AKT in cells was detected by Western blot. The results showed that, despite having no significant effect on total AKT expression when compared to the control, SC79 significantly reversed the inhibitory effect of NEFA on the expression of p-AKT (*p* < 0.01, Figure 5G,H).

### 3.6. AKT Activator SC79 Reduced the Sensitivity of BGCs to NEFA-Mediated Oxidative Stress

**Figure 6:**
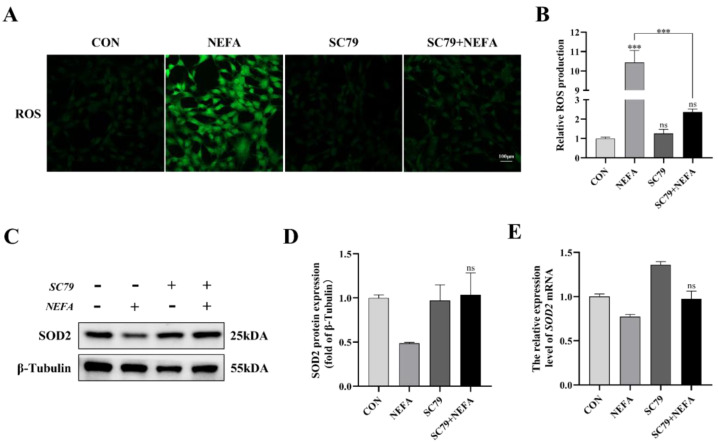
AKT activator SC79 alleviated NEFA−induced ROS and oxidative stress in BGCs. Experiments were performed with non−treated, 5 mg/mL SC79, 1.2 mM NEFA, or 5 mg/mL SC79 for 2 h followed by 1.2 mM NEFA supplement for 24 h. (**A**,**B**) ROS formation was determined by fluorescence microscopy. Green: DCFH−DA, scale bar: 100 µm. (**C**–**E**) SOD2 protein and gene expression in BGCs. The quantification of the relative protein levels (performed using Image J) is shown under the band (protein/β−Tubulin). This experiment was repeated 3 times, and data are expressed as mean ± standard errors of the mean (SEM), *** *p* < 0.001; ns, not significant.

The cell’s ROS levels were measured with immunofluorescence microscopy. As depicted in Figure 6, SC79 pretreatment could significantly reduce ROS production in cells following NEFA treatment (*p* < 0.001, Figure 6A,B). The expression level of SOD2 was measured by Western blot and qRT-PCR. There was no significant difference between NEFA and SC79+NEFA groups, but pretreatment of SC79 had a tendency to reverse the reduction of SOD2 caused by NEFA addition (*p* > 0.05, Figure 6C–E). These findings suggested that AKT activator SC79 may reduce NEFA-induced oxidative stress in BGCs.

### 3.7. AKT Activator SC79 Inhibited NEFA-Mediated Apoptosis in BGCs

**Figure 7:**
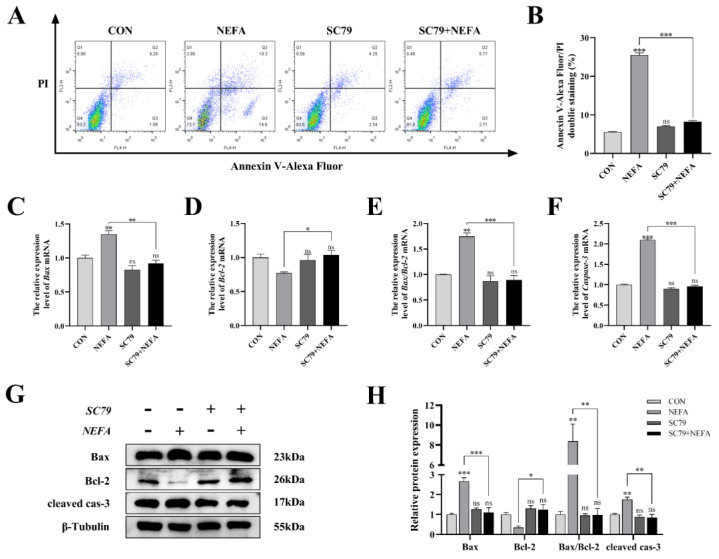
AKT activator SC79 decreased NEFA−induced apoptosis in BGCs. Experiments were performed after NEFA treatment of BGCs for 24 h with or without SC79 pretreatment for 2 h. (**A**,**B**) Flow cytometry detected the apoptosis of BGCs. (**C**–**F**) The mRNA levels of Bax, Bcl−2, and Caspase−3 were determined using qRT−PCR. (**G**,**H**) The apoptosis−related proteins Bax, Bcl−2, and Caspase−3 were analyzed by Western blot. The quantification of the relative protein levels (performed using Image J) is shown under the band (protein/β−Tubulin). This experiment was repeated 3 times, and data are expressed as mean ± standard errors of the mean (SEM), * *p* < 0.05; ** *p* < 0.01; *** *p* < 0.001; ns, not significant.

Apoptosis was significantly reduced in BGCs by the pathway activator (*p* < 0.001, Figure 7A,B) using flow cytometry in comparison to only NEFA-treated groups. SC79 pro-treatment significantly reversed the significant increase in Bax and Caspase-3 mRNA and protein expression caused by NEFA (*p* < 0.05, Figure 7C–H). Along these lines, the ratio of Bax to Bcl-2 was significantly reduced (*p* < 0.01, Figure 7E,H) in SC79-treated cells, contrasted with the NEFA-only group. Taken together, activation of the PI3K/AKT pathway inhibited NEFA-induced apoptosis in BGCs.

### 3.8. AKT Activator SC79 Reversed the Effects of NEFA on Steroid Hormone Synthesis Gene Expression in BGCs

**Figure 8:**
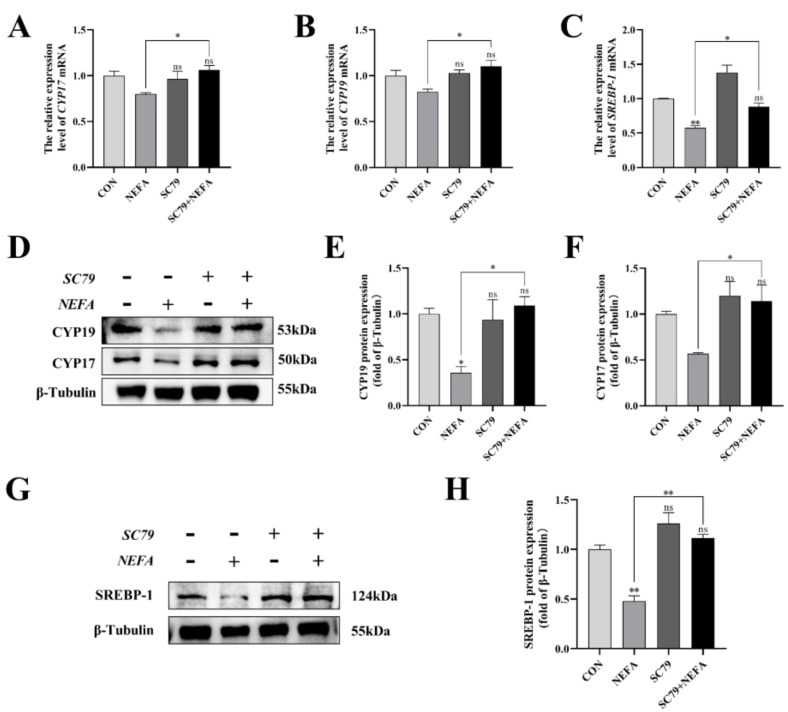
Effects of AKT activator SC79 on NEFA−induced steroid hormone synthesis gene expression in BGCs. Experiments were performed after NEFA treatment of BGCs for 24 h with or without SC79 pretreatment for 2 h. (**A**,**B**,**D**–**F**) CYP17 and CYP19 protein and gene expression in BGCs under treatment with AKT activator SC79. (**C**,**G**,**H**) SREBP−1 protein and gene expression in BGCs. The quantification of the relative protein levels (performed using Image J) is shown under the band (protein/β−Tubulin). This experiment was repeated 3 times, and data are expressed as mean ± standard errors of the mean (SEM), * *p* < 0.05; ** *p* < 0.01; ns, not significant.

This experiment measured the level of expression of the steroid synthesis and regulation-related enzyme genes in cells to better understand how NEFA affected BGCs steroid hormone secretion. The CYP17, CYP19, and SREBP-1 levels of the SC79+NEFA group were significantly higher than those of the NEFA group (*p* < 0.05), and they were not significantly different from those of the control group with no treatment (*p* > 0.05, Figure 8A–H). It was possible to draw the conclusion that NEFA’s changes to hormone-related genes in BGCs could be reversed by SC79.

### 3.9. NEFA Regulated FoxO1 Levels and Activity via PI3K/AKT Signaling Pathway in BGCs

**Figure 9:**
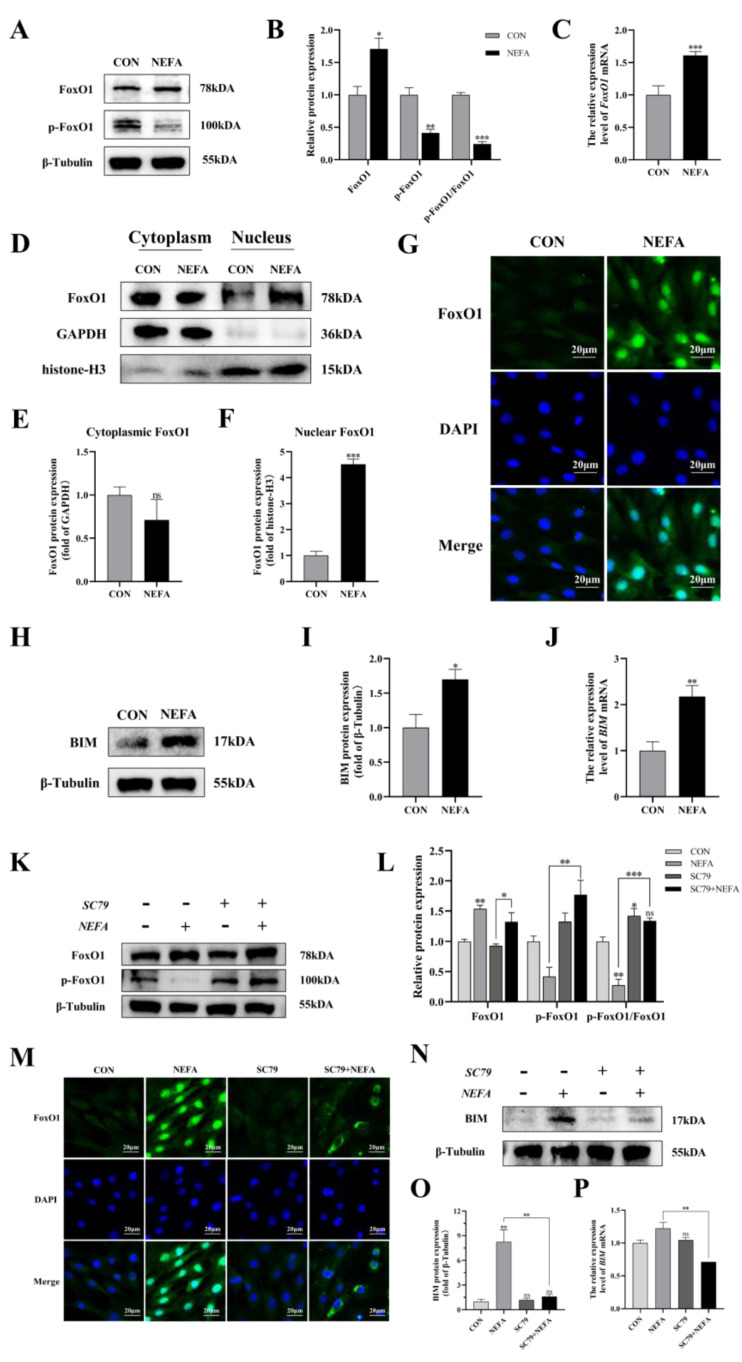
Regulation of FoxO1 levels, activity, and translocation by NEFA and AKT activator SC79 in BGCs. Experiments of (**A**–**J**): BGCs were treated with or without 1.2 mM NEFA for 24 h. Experiments of (**K**–**P**) were performed after NEFA treatment of BGCs for 24 h with or without SC79 pretreatment for 2 h. (**A**–**C**,**K**,**L**) Protein levels of FoxO1 and FoxO1 phosphorylation (Ser256) were analyzed by Western blot and mRNA levels of FoxO1 were tested by qRT−PCR. (**D**–**F**) Western blot results and analysis of FoxO1 in nucleus and cytosol. (**G**,**M**) Immunofluorescence staining of FoxO1 nuclear translocation Green: DCFH−DA, Blue: DAPI, scale bar: 20µm. (**H**–**J**,**N**–**P**) mRNA and protein expression of BIM. The quantification of the relative protein levels (performed using Image J) is shown under the band (protein/(β−Tubulin/GAPDH/histone−H3)). This experiment was repeated 3 times, and data are expressed as mean ± standard errors of the mean (SEM), * *p* < 0.05; ** *p* < 0.01; *** *p* < 0.001; ns, not significant.

To determine whether the FoxO1 transcription factor affected BGCs’ capacity to add NEFA, we examined FoxO1 expression and translocation. Under NEFA treatment, it decreased FoxO1 phosphorylation in comparison to the control group but increased total FoxO1 expression (*p* < 0.01, Figure 9A–C). The levels of FoxO1 in the nucleus significantly increased following the addition of NEFA (*p* < 0.001), but there were no significant changes in the cytoplasmic levels in comparison to the control group (*p* > 0.05, Figure 9D–G). In addition, the supplement of NEFA significantly increased the expression of BCL-2 interacting mediator of cell death (BIM), an apoptosis-related factor downstream of FoxO1 (*p* < 0.05, Figure 9H–J). These findings demonstrated that FoxO1 may play a significant role in the detrimental effects of NEFA on BGCs.

Next, the effect of PI3K/AKT activator SC79 on FoxO1 expression in the cells was examined. The fact that the ratio of p-FoxO1/FoxO1 in the SC79+NEFA group was significantly higher than that in the NEFA-treated groups (*p* < 0.001, Figure 9K,L), suggested that the phosphorylation of FoxO1 in cells was facilitated by the activation of p-AKT by SC79. As depicted in Figure 9M, the expression of FoxO1 decreased in the nucleus while increasing in the cytoplasm under SC79 exposure compared with the cells only treated by NEFA. Moreover, SC79 pretreatment significantly reduced the NEFA-induced increase in BIM expression (*p* < 0.01, Figure 9N–P). Overall, this suggested that NEFA may promote FoxO1 expression and nuclear localization by inhibiting the PI3K/AKT pathway.

### 3.10. Establishment of an in Vitro Knockdown of FoxO1

**Figure 10:**
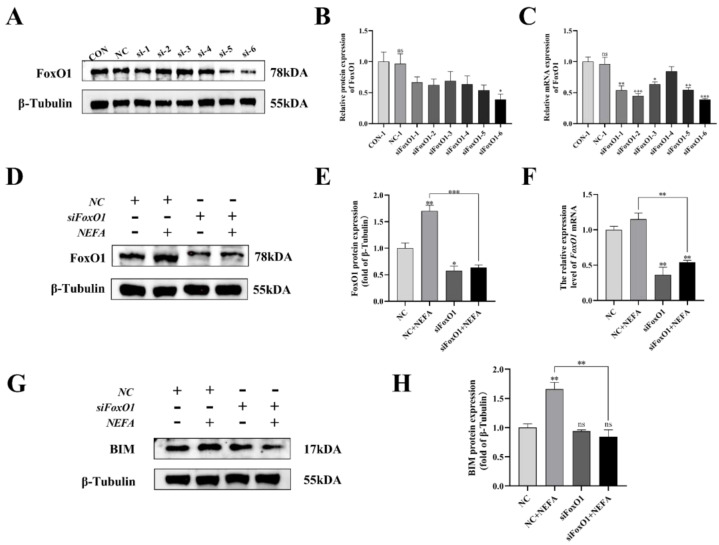
Screening and validation of si−RNA of FoxO1. BGCs were transfected with NC or siFoxO1 for 6 h before being treated with NEFA for 24 h. (**A**–**C**) Screening of si−FoxO1 by qRT−PCR and Western blot. (**D**–**F**) The expression of FoxO1 was determined by FoxO1 siRNA treatment. (**G**,**H**) Protein expression of BIM by FoxO1 siRNA treatment. The relative protein levels (performed using Image J) are quantified under the band (protein/β−Tubulin). This experiment was repeated 3 times, and data are expressed as mean ± standard errors of the mean (SEM), * *p* < 0.05; ** *p* < 0.01; *** *p* < 0.001; ns, not significant.

To further explore the mechanism by which NEFA contributes to FoxO1’s negative effects on BGCs, this study generated six pairs of FoxO1 sequences (siFoxO1) and tested their knockdown efficiency. The mRNA and protein levels of FoxO1 decreased in varying degrees when compared to the control group (Figure 10A–C). As a result, the siFoxO1 (siFoxO1-6) with higher knockdown efficiency was utilized for subsequent experiments. We indicated that FoxO1 expression levels in the siFoxO1+NEFA treatment group decreased significantly in comparison to the NEFA-only group (*p* < 0.01, Figure 10D–F), and FoxO1 knockdown markedly decreased the accumulation of BIM after NEFA treatment (*p* < 0.01, Figure 10G,H). Collectively, these results indicated that siFoxO1 could significantly inhibit the expression of FoxO1 and its downstream BIM in cells, which could be used in subsequent studies.

### 3.11. Silencing of FoxO1 by siRNA Reversed the Pro-Apoptotic Phenomenon Induced by the Addition of NEFA to BGCs

**Figure 11:**
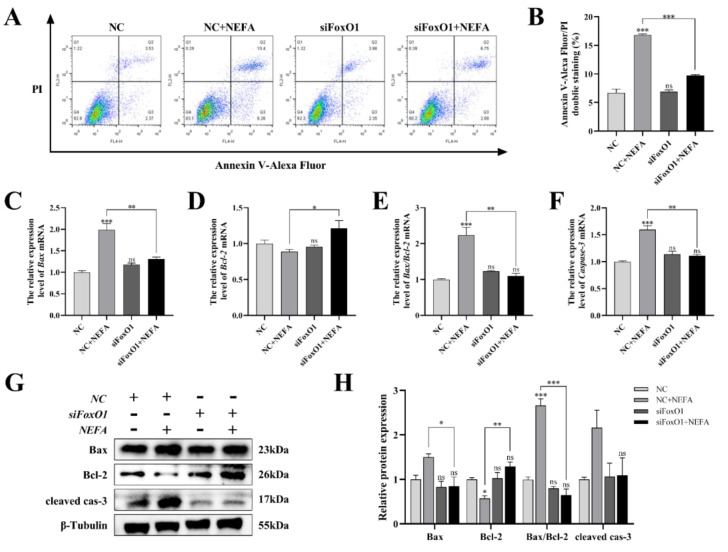
Effects of the si−FoxO1 on NEFA−induced apoptosis in BGCs. BGCs were transfected with NC or siFoxO1 for 6 h before being treated with NEFA for 24 h. (**A**,**B**) Flow cytometry detected the apoptosis of BGCs after transfection with siFoxO1/siNC. (**C**–**F**) The qRT−PCR showed relative expression of Bax, Bcl−2, and Caspase−3. (**G**,**H**) Immunoblotting was performed to detect the protein expression of Bax, Bcl−2, and Caspase−3. The quantification of the relative protein levels (performed using Image J) is shown under the band (protein/β−Tubulin). This experiment was repeated 3 times, and data are expressed as mean ± standard errors of the mean (SEM), * *p* < 0.05; ** *p* < 0.01; *** *p* < 0.001; ns, not significant.

Next, to determine whether FoxO1 is involved in NEFA-induced apoptosis, we first used flow cytometry to examine cellular apoptosis. FoxO1 siRNA transfection significantly reduced NEFA-induced cellular apoptosis, as depicted in Figure 11A,B (*p* < 0.001). In the siFoxO1+NEFA groups, the expression of Bax and the ratio of Bax/Bcl-2 were significantly decreased (*p* < 0.05), and the expression of cleaved caspase-3 also tended to decrease, while the expression of Bcl-2 was significantly increased (*p* < 0.05, Figure 11C–H) compared with siNC+NEFA group, indicating that the situation of apoptosis was alleviated. The silence of FoxO1 caused by siRNA could inhibit the expression and activation of downstream apoptosis-related target genes, thus protecting the BGCs from NEFA-induced injury. These results pointed to a central role for FoxO1 in NEFA-induced apoptosis.

### 3.12. FoxO1 Regulated the Effects of NEFA on Steroid Hormone Synthesis Gene Expression in BGCs

**Figure 12:**
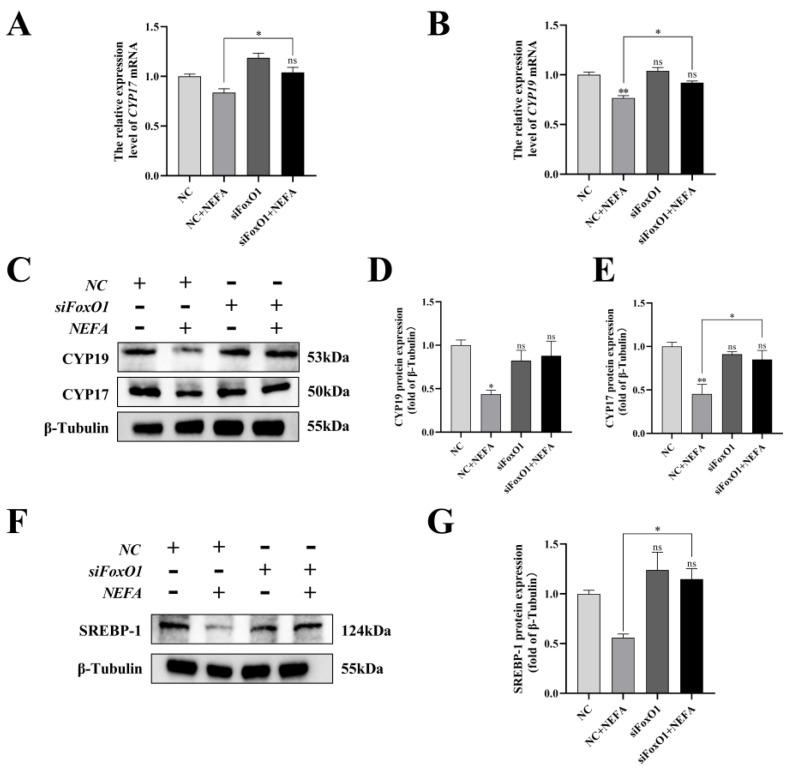
Effects of the si−FoxO1 on NEFA−induced steroid hormone synthesis gene expression in BGCs. BGCs were transfected with NC or siFoxO1 for 6 h before being treated with NEFA for 24 h. (**A**–**E**) CYP17 and CYP19 protein and gene expression in BGCs under treatment with siFoxO1/siNC. (**F**,**G**) SREBP−1 protein expression in BGCs after the silence of FoxO1. The quantification of the relative protein levels (performed using Image J) is shown under the band (protein/β−Tubulin). This experiment was repeated 3 times, and data are expressed as mean ± standard errors of the mean (SEM), * *p* < 0.05; ** *p* < 0.01; ns, not significant.

To determine the role of FoxO1 in NEFA-induced steroid hormone secretion, the protein contents of related proteins (CYP17, CYP19, SREBP-1) were determined by immunoblotting. The results revealed that silencing of FoxO1 before NEFA addition led to an upregulation of intracellular CYP19 expression and a significant increase in CYP17 and SREBP-1 protein expression compared to the NEFA-only group (*p* < 0.05, Figure 12). Similar results were also obtained via qRT-PCR. This suggested that silencing FoxO1 may reverse the effects of NEFA on hormone secretion in BGCs.

## 4. Discussion

The high level of circulating NEFA may lead to the decline of the reproductive performance of dairy cows [5]. To date, reports on the effects of NEFA on BGCs and their mechanism of action are not comprehensively reported. Therefore, in this experiment, we explored the negative effects of NEFA in BGCs in terms of proliferation, oxidative stress, apoptosis, and steroid hormone synthesis. The AKT activator SC79, as well as the knockdown of FoxO1, suggested that NEFA may act by affecting AKT activity and nuclear translocation of FoxO1. The results of this study suggested that PI3K/AKT/FoxO1 may be a key pathway to study NEFA-induced oxidative damage and apoptosis in BGCs.

Lipid mobilization caused by NEB can easily trigger oxidative stress response [27]. The results of this experiment showed that NEFA increased intracellular ROS levels, which is consistent with a previous study in rat pheochromocytoma cells [28]. MDA is a toxic end product of lipid peroxidation reactions after ROS degradation [29]. The antioxidant enzyme system is the first line of defense against oxidative injury. Among them, the level of expression of SOD2, which is mainly present in mitochondria, the main site of ROS production, directly reflects the strength of antioxidant capacity [30]. A previous study has shown that NEFA treatment induced oxidative stress in BRL-3A rat liver cells by altering the activity of MDA and endogenous antioxidants [31]. Our data showed that the addition of NEFA significantly increased the content of MDA and down-regulated the activity of antioxidant enzymes SOD2. This indicated that excess NEFA induced oxidative stress in BGCs.

High concentrations of NEFA can restrain cell growth [32]. In this study, we found that NEFA significantly inhibited cell proliferation and increased the apoptosis rate of BGCs in a concentration-dependent manner. Bcl-2 and Bax are important regulators of apoptosis. When Bax is overexpressed relative to Bcl-2, the caspase-mediated apoptotic cascade response will be activated [33]. It was shown that the additive amount of NEFA induced apoptosis in bovine mammary epithelial cells in a dose-dependent manner by increasing the expression of apoptotic genes [34]. Our results suggested that the NEFA-treated group increased Bax, Bax/Bcl-2, and Caspase-3 levels significantly, further confirming that NEFA caused the generation of apoptosis in BGCs.

Ovarian granulosa cells play an important role in follicular growth and development, and their status is closely related to the reproductive capacity of animals [35]. The synthesis of steroid hormones is a key biological process in the development of the follicle [36]. SREBP-1 can mediate endocrine hormone regulatory processes by regulating the synthesis and metabolism of cholesterol and fatty acids, precursors of steroids [37]. StAR is responsible for transporting cholesterol across the mitochondrial membrane, which is later converted to pregnenolone by CYP11 and finally to progesterone by the HSD3β [38]. In our study, the addition of NEFA decreased the expression levels of SREBP-1 and StAR and upregulated the expression of CYP11 and HSD3β1 in BGCs. It can be hypothesized that NEFA restricted the production and transport of cholesterol, but promoted the conversion of cholesterol to progesterone. However, Lima et al. [39] found that high levels of NEFA in the follicular fluid of dairy cows increased levels of StAR expression and decreased CYP11 expression. Their samples had cystic ovarian follicles, and the different reasons from our study may be related to this. The potential mechanisms by which NEFA affects genes related to steroid hormone synthesis still need further investigation. Cytochrome p450 family is important for ovarian hormone production, follicle growth, and maturation for ovulation [40]. CYP17 is involved in the regulation of physiological processes related to androgen synthesis and metabolism in living organisms, and CYP19 is a key rate-limiting enzyme for estrogen synthesis [41]. They regulate folliculogenesis, development, and atresia at different times by affecting the synthesis of steroid hormones [42]. We found that CYP17 and CYP19 were significantly downregulated after NEFA stimulation in BGCs, indicating that steroid hormone production was negatively affected. The above results indicated that NEFA could profoundly disturb the normal synthesis of hormones in BGCs by causing dysregulation of the expression of key enzyme genes in steroid hormone synthesis, thus affecting the growth and development of follicles. Ferst et al. [43] found that the addition of NEFA through follicular injection in cows increased the negative impact of follicular development, which supports our results.

The PI3K/AKT signaling pathway is involved in constituting an intracellular homeostatic signaling network, and its functions include and are not limited to influencing cell proliferation, differentiation, metabolism, and autophagy [44,45]. A study by Sun et al. [46] in the human hepatic carcinoma cell line found that the addition of NEFA induced insulin resistance by inhibiting the PI3K/AKT signaling pathway. The effects of the PI3K/AKT signaling pathway in NEFA-mediated BGCs still need further investigation. In the present study, it was demonstrated that the addition of NEFA significantly reduced the expression levels of PI3K and p-AKT compared with normal controls, indicating that NEFA influenced the activation of the PI3K/AKT signaling pathway in BGCs. To clarify whether the negative effects of NEFA on BGCs result from inhibition of the PI3K/AKT signaling pathway, we used SC79, a specific activator of AKT, for validation. First, we demonstrated that SC79 triggered phosphorylation of the AKT S473 site and could block the inhibitory effect of NEFA on p-AKT. It was reported that SC79 would inhibit dexamethasone-induced oxidative stress and cell death by activating AKT and downstream factors, as well as reversing the accumulation of ROS, in primary and MC3T3-E1 osteoblasts of mice [47]. Our findings confirmed that SC79 pretreatment significantly alleviated the increase in ROS and had a tendency to alleviate the downregulation of SOD2 caused by NEFA addition. This indicated that the pretreatment of SC79 could inhibit the occurrence of oxidative stress. The previous findings in regard that SC79 would significantly alleviate TNF-α-induced apoptosis in both the cultured human hepatocellular carcinoma cells and the primary mouse hepatocytes through activation of AKT [48]. The relationship between PI3K/AKT signaling pathway activation and NEFA-induced granulosa cell apoptosis was further investigated in this study. Our data showed that SC79 pretreatment significantly suppressed the expression of Bax and Caspase-3, as well as the ratio of Bax to Bcl-2, suggesting that the activation of the PI3K/AKT pathway could effectively alleviate the NEFA-induced apoptotic damage in BGCs. In terms of steroid hormones, inhibition of the PI3K/AKT signaling pathway in murine primary hepatocytes suppresses the expression of cytochrome P4502D22 [49]. We confirmed that the addition of SC79 significantly upregulated the expression levels of SREBP-1, CYP17, and CYP19 in BGCs compared to the group with NEFA addition only, indicating that SC79 pretreatment effectively reversed the negative effects of NEFA on endocrine hormones in BGCs. Taken together, it can be implied that NEFA caused damage to cells by inhibiting PI3K/AKT signaling pathway, such as promoting oxidative stress and cell apoptosis, thus affecting hormone secretion, while AKT activator SC79 could reverse these phenomena and alleviate the negative effects caused by NEFA.

FoxO1, a key PI3K/AKT downstream target, has a close association with normal ovarian development [50]. Shukla et al. [51] found that berberine significantly upregulated the expression of FoxO1 and its downstream pro-apoptotic target gene BIM, leading to HepG2 cell damage. Our results showed that under the stimulation of NEFA, the levels of FoxO1 and BIM increased significantly in cells. FoxO1 may be involved in the negative effects caused by NEFA on BGCs. Inhibition of FoxO1 production may alleviate apoptosis or dysfunction [52,53]. Previous studies suggested that FoxO1 knockdown protected human cancer cells against death caused by trichostatin A [54]. Similarly, our further results showed that the knockdown of FoxO1 significantly reduced Annexin-V/PI double-staining rate, pro-apoptotic factor BIM, and Bax expression levels compared with siNC+NEFA group, and anti-apoptotic factor Bcl-2 showed the opposite trend. We contended that NEFA-induced apoptosis in BGCs is closely related to FoxO1. Meanwhile, in terms of steroid hormones, it has been demonstrated in goat mammary epithelial cells that FoxO1 inhibits insulin-induced SREBP1 activity [55]. Our data demonstrated that knockdown of FoxO1 before NEFA-treated cells, caused the expression of SREBP-1 and CYP17 to be significantly increased, and CYP19 also showed an increasing trend. This suggested that the FoxO1-knockdown inhibited the transcriptional activation of its downstream apoptosis-related target genes and reversed the effects of NEFA addition on steroid synthesis genes. In summary, the following conclusion can be drawn: FoxO1 is a key molecule in the PI3K/AKT pathway involved in NEFA-induced apoptosis in BGCs.

The transcriptional activity and subcellular localization of FoxO1 are closely related to the regulation of its protein phosphorylation level [56]. We found that NEFA treatment decreased the p-FoxO1/FoxO1 ratio and promoted the FoxO1 translocation into the nucleus in BGCs. Similarly, Lee H and Lee J [57] found that the addition of FFA inhibited the phosphorylation of PI3K, AKT, and FOXO1 in HepG2 cells. Activation of the PI3K/AKT signaling pathway mediates FoxO1 phosphorylation. Moeinifard et al. [58] found in human pancreatic cancer cells that Britannin induced an increase in FOXO1 content in the nucleus by impairing AKT phosphorylation through the accumulation of ROS. Several previous studies also reported a similar effect of AKT activation on FoxO1 phosphorylation and nuclear translocation [59,60]. Our study showed that the activation of p-AKT by SC79 promoted the phosphorylation of FoxO1 in cells, thereby inducing the FoxO1 from the nucleus to the cytoplasm, impairing its transcriptional activity and ultimately inhibiting the expression of its downstream target apoptotic genes. It was demonstrated that the PI3K/AKT pathway was involved in the negative effects of NEFA induction in BGCs through the regulation of FoxO1 phosphorylation and nuclear translocation. These further suggested that NEFA may promote apoptosis in BGCs by inhibiting the PI3K/AKT signaling pathway, reducing AKT activity to increase nuclear accumulation and transcriptional activity of FoxO1, thus affecting follicle growth and development.

## 5. Conclusions

In conclusion, this study found that NEFA negatively affected BGCs by affecting the PI3K/AKT/FoxO1 signaling pathway. NEFA increased intracellular FoxO1 levels and caused oxidative stress through inhibition of PI3K and p-AKT activity, resulting in dephosphorylation and massive nuclear translocation of FoxO1 to perform transcriptional functions. Then, the downstream apoptotic pathway was activated, causing apoptosis in BGCs and affecting the synthesis of cellular steroid hormones, which may affect the growth, development, and atresia of follicles. A better understanding of the mechanism of the PI3K/AKT/FoxO1 pathway under the action of NEFA and its mediated apoptotic pathway provides a new perspective on how to maintain a stable intracellular environment, normalize ovarian function, and improve dairy cows’ reproductive performance. Further exploration of the specific drugs and molecular mechanisms that activate this signaling pathway could provide insight into how these factors mitigate the negative effects of NEFA for practical application in production.

## Figures and Tables

**Table 1:** Primer sequences of mRNA in this experiment for qRT-PCR analysis.

Genes	Forward (5′-3′)	Reverse (5′-3′)	Accession Number
SOD2	TGAGCCCTAACGGTGGTGGA	ATTGAAGCCGAGCCAACCCC	NM_201527.2
NQO1	CCGAGGATGCCCGAGTTCAG	ACTTGCCCAAGTGATGGCCC	NM_001034535.1
HMOX1	CAGGCACCTCTCCTGCGATG	AGACAGCAGGAGCCCTGACA	NM_001014912.1
Bax	CATCATGGGCTGGACATTGG	AAGATGGTCACTGTCTGCCA	NM_173894.1
Bcl-2	ATGACCGAGTACCTGAACCG	GCCATACAGCTCCACAAAGG	NM_001166486.1
Caspase-3	TTGAGACAGACAGTGGTGCT	TCTTTGCATTTCGCCAGGAA	NM_001077840.1
SREBP-1	CCTGTGGGCCACCGTTTCTT	CCACGCAATTCAGTGCTCGC	NM_001113302.1
HSD3β1	AACCCTTGCTTCAACCGCCA	CCCAGGCTTGTGTCCGTGTT	NM_174343.3
StAR	TGACGTGGGCAAGGTGTTCC	ATGCCAGCCAGCACACACAT	NM_174189.3
CYP11	AGACTTGGAGGGACCATGTA	GGATGCCTGGGTAATTCCTAAA	NM_176644.2
CYP17	CTGCTGCCACCCAGACACAA	GGCCAACTGGTGGTGTCCAA	NM_174304.2
CYP19	GACGCTTCCACGTGCAGACT	GGGGCAGGGACTGACCAAAC	NM_174305.1
PI3K	TTGCCGTGCATGTGGGATGT	GGTCGCCGCATTTGCTCAAC	NM_174574.1
AKT	CGTTCTCCAGAACTCCCGGC	GGTAATCCAGGGCCGACACG	NM_173986.2
FoxO1	CGACTCACGCTGTCGCAGAT	GCACGCGGATGAACTTGCTG	XM_025000053.1
BIM	TGCGTCACCAGGCAGTTGAG	CTGCCCACCCTTCCAGCAAA	NM_001075310.1
β-actin	GGGCAGGTCATCACCATCGG	TCATTGTGCTGGGTGCCAGG	NM_173979.3

**Table 2:** Antibodies used in this experiment for Western blot.

Antibody	Host Species	Dilution	Vendor	Catalog Number
SOD2	rabbit	1:500	ABclonal Technology, China	A1340
Bax	rabbit	1:2000	Proteintech, China	50599-2-Ig
Bcl-2	rabbit	1:500	Wanleibio, China	WL01556
Cleaved Caspase-3	rabbit	1:500	Affinity, China	AF7022
SREBP-1	rabbit	1:1000	Proteintech, China	14088-1-AP
CYP17	rabbit	1:500	ABclonal Technology, China	A5067
CYP19	rabbit	1:500	ABclonal Technology, China	A2161
AKT	rabbit	1:500	Bioss, China	bs-0115R
p-AKT	rabbit	1:500	ABclonal Technology, China	AP0637
PI3K	rabbit	1:500	ABclonal Technology, China	A0265
FoxO1	rabbit	1:1000	Cell Signalling Technology, USA	#2880
p-FoxO1	rabbit	1:500	ABclonal Technology, China	AP0172
BIM	rabbit	1:500	ABclonal Technology, China	A15771
GAPDH	rabbit	1:5000	Proteintech, China	10494-1-AP
histone-H3	rabbit	1:2000	Proteintech, China	17168-1-AP
β-Tubulin	rabbit	1:5000	Bioworld Technology, USA	AP0064

## Data Availability

The data presented in this study are included within the article.
